# OBP14 (Odorant-Binding Protein) Sensing in *Adelphocoris lineolatus* Based on Peptide Nucleic Acid and Graphene Oxide

**DOI:** 10.3390/insects12050422

**Published:** 2021-05-08

**Authors:** Wenhua Tian, Tao Zhang, Shaohua Gu, Yuyuan Guo, Xiwu Gao, Yongjun Zhang

**Affiliations:** 1 Department of Entomology, College of Plant Protection, China Agricultural University, Beijing 100193, China; jane_happy08@163.com (W.T.); gushaohua@cau.edu.cn (S.G.); gaoxiwu@263.net.cn (X.G.); 2 State Key Laboratory for Biology of Plant Diseases and Insect Pests, Institute of Plant Protection, Chinese Academy of Agricultural Sciences, Beijing 100193, China; zhangtao0372@126.com (T.Z.); yuyuanguo@hotmail.com (Y.G.)

**Keywords:** *Adelphocoris lineolatus*, *AlinOBP14*, graphene oxide (GO), in vitro, in vivo, juvenile hormone, mRNA biosensor, peptide nucleic acid (PNA), Plus-C OBPs, terpenoids

## Abstract

**Simple Summary:**

The alfalfa plant bug, *Adelphocoris lineolatus* (Goeze) (Hemiptera: Miridae), is a serious agricultural pest in China. Previous studies have shown that olfaction plays a crucial role in the survival and reproduction of herbivorous insects, such as *A. lineolatus,* and OBPs (odorant-binding proteins) are involved in the initial steps of olfaction. Due to the complex intracellular environment in insects, detection of the expression of OBP genes by conventional methods is limited in vitro. In this study, we developed a PNA-GO- (peptide nucleic acid and graphene oxide) based mRNA biosensor that allows detection of the expression of the *AlinOBP14* gene in *A. lineolatus*. We demonstrate that *AlinOBP14* is expressed in the adult head, which is consistent with previous research on the expression profiles of *AlinOBP14* in *A. lineolatus* using quantitative real-time PCR. The results demonstrate that the method we developed is both feasible and reliable. In addition, this method exhibited good stability and specificity in detection of chemically synthesized targets in vitro as well as the expression of the target OBP gene in vivo.

**Abstract:**

OBPs play a crucial role in the recognition of ligands and are involved in the initial steps of semiochemical perception. The diverse expression of OBP genes allows them to participate in different physiological functions in insects. In contrast to classic OBPs with typical olfactory roles in *A. lineolatus,* the physiological functions of Plus-C OBPs remain largely unknown. In addition, detection of the expression of insect OBP genes by conventional methods is difficult in vitro. Here, we focused on *AlinOBP14*, a Plus-C OBP from *A. lineolatus,* and we developed a PNA-GO-based mRNA biosensor to detect the expression of *AlinOBP14*. The results demonstrated that *AlinOBP14* plays dual roles in *A. lineolatus*. The *AlinOBP14* is expressed beneath the epidermis of the vertex and gena in heads of *A. lineolatus,* and it functions as a carrier for three terpenoids, while *AlinOBP14* is also expressed in the peripheral antennal lobe and functions as a carrier for endogenous compounds such as precursors for juvenile hormone (JH) and JHⅢ. Our investigation provides a new method to detect the expression of OBP genes in insects, and the technique will facilitate the use of these genes as potential targets for novel insect behavioral regulation strategies against the pest.

## 1. Introduction

Olfaction, an important chemosensory modality in insects, plays a crucial role in sensing chemical signals from the environment [1,2]. Insects use a range of environmental cues to locate their mates, host plants, and oviposition and hibernation sites, Olfaction is also employed to avoid predators, unsuitable hosts, or dangerous situations [3]. Insect odorant-binding proteins (OBPs), as ligand carriers or solubilizers in lymph, contribute to insect-specific olfactory perception [4]. The diverse tissue expression of OBP genes allows them to participate in different physiological functions [5]. Detection of the expression of OBP genes is central to elucidating the molecular basis of olfactory reception in insects and to facilitating the use of OBP genes as potential targets for interference with pest olfactory recognition.

The alfalfa plant bug, *Adelphocoris lineolatus* (Goeze) (Hemiptera: Miridae), has become a serious pest in transgenic Bt cotton fields in China [6,7,8]. Thirty-four putative OBP genes have been identified in *A. lineolatus*, including the classic OBP subfamily and the Plus-C OBP subfamily [9]. In the present study, we analyzed the expression of the *AlinOBP14* gene, a Plus-C OBP gene from *A. lineolatus*. The aims of this study were to identify the physiological roles of *AlinOBP14* in *A. lineolatus* and to facilitate the implementation of the OBP gene as a potential target for designing behavior control strategies to effectively manage the pest.

At present, real-time quantitative PCR (RT-qPCR) is widely used for analyzing relative expression levels of OBP genes in insects [10,11,12]. Although RT-qPCR is an efficient technique for quantifying expression levels of targeted genes during various biological process, multiple factors, including the integrity of RNA, the efficiency of the designed primers, the stability of the reference gene, the efficiency of cDNA synthesis, and PCR efficiency, can significantly influence experimental results [13,14]. In other words, obtaining reliable and accurate experimental data depend on specific experimental conditions. Northern blots are also used to explore the functions of OBPs in insects and to analyze RNA extracted from various developmental stages [15]. However, the hybridization and blotting-based Northern analyses are laborious, intricate, and always suffer from disadvantages such as large RNA requirements, sample degradation, and environmental contamination related to the probe labeling. Although microarray hybridization experiments can enable examination of the relative expression levels of the OBP transcripts in tissues associated with chemosensory perception [16], the high cost, the large amounts of microarray data analysis, and the requirement for extremely innovative statistical techniques and bioinformatic tools limit its widespread application. Currently, fluorescence in situ hybridization (FISH) is the prevailing method for analyzing the distribution features of OBPs in insects [17]. However, this intricate and laborious method suffers from low sensitivity and time-consuming operation. Thus, a fast and simple method for the detection of the expression levels of OBP genes in insects is urgently needed. Coupling nanomaterials with biomolecular recognition events represents a new direction in nanotechnology toward the development of novel biomolecular detection techniques.

GO is a single-atom-thick carbon material and a water-soluble derivative of graphene due to the presence of suspended hydroxyl and carboxylic groups at the surface [18]. Single-stranded nucleic acids can be strongly adsorbed on the surface by π-π stacking interactions and hydrogen bonds. However, the interactions between double-stranded nucleic acids and GO are rather weak [19]. Additionally, the fluorescence signals of dye-labeled nucleic acid probes are quenched by the addition of GO due to the strong hydrophobic adsorption of the probes at the GO surface and highly efficient long-range nanoscale energy transfer from the dye to GO, whereas the quenched fluorescence signal is recovered while the dye-labeled nucleic acids probe hybridized with complementary target mRNA and desorbed from the GO surface [20]. 

We designed three PNA probes conjugated with three fluorescent dyes complementary to three regions of target mRNA (probes: CY5-P1, ROX-P2, and FAM-P3; target mRNA: AlinOBP14-T1, AlinOBP14-T2, and AlinOBP14-T3) to detect chemically-synthesized target mRNA in vitro and the expression of the *AlinOBP14* gene in *A. lineolatus* (Supporting information Figure S1). 

In this study, we report a PNA-GO-based mRNA biosensor that allows simple, specific detection of the expression of the *AlinOBP14* gene in *A. lineolatus*. The biosensor we developed allows monitoring of three regions of the target mRNA in *A. lineolatus* without cross-reactivity. We found that *AlinOBP14* is highly expressed in the adult head of *A. lineolatus,* consistent with previous research on the expression profiles of *AlinOBP14* in *A. lineolatus* by quantitative real-time PCR [21]. The results suggest that the method we developed is both feasible and reliable. Owing to the good stability and specificity for the detection of the target mRNA in *A. lineolatus*, we conclude that the method we developed can be applied to detect the expression of mRNA in insects.

## 2. Materials and Methods

### 2.1. Materials and Reagents

GO (XF020, 100690) was purchased from XFNANO Materials Tech Co., Ltd. (Nanjing, China). The mRNA used in the study was synthesized by Sangon Biotech Co., Ltd. (Shanghai, China). Each PNA probe was labeled with a different fluorescent dye; these were obtained from Sangon Biotech Co., Ltd. (Shanghai, China). The probes were purified by HPLC. All PNA and mRNA sequences are listed in Supporting Information Table S1 (HPLC profiles and MS spectra for all the PNA derivatives are shown in Supporting Information Figure S2).

### 2.2. Insect Rearing and Collection

The *A. lineolatus* adults were collected from cotton fields at the Lang Fang Experimental Station of the Chinese Academy of Agricultural Sciences, Hebei Province, China. In the laboratory, the colony was reared on green beans (*Phaseolus vulgaris* L.) and 10% sucrose solution. The laboratory colony was kept in plastic containers (20 cm × 12 cm × 8 cm) and maintained at 30 °C, 60 ± 5% relative humidity (R.H.), with a 14:10 light: dark (L:D) photoperiod [22]. 

### 2.3. Characterization of GO

Fourier transform infrared (FT-IR) measurement of GO powder was carried out with a NICOLET IS10 FTIR spectrometer (Thermo Electron Corp., Madison, WI, USA) by the KBr pellet method (GO:KBr = 1:100). Raman spectra were obtained using a DXR Raman Microscope (Thermo Fisher Scientific, Waltham, MA, USA) with an excitation wavelength of 514 nm. The X-ray diffraction (XRD) patterns were recorded on a Bruker D8 X-ray diffractometer (Bruker Co., Berlin, Germany). Atomic force microscopy (AFM) images were obtained by using a SPM 9700 microscope (Shimadzu, Kyoto, Japan). The samples were prepared by depositing dispersions of GO on a freshly cleaved mica surface. The images were flattened and analyzed using the Gwyddion software. 

### 2.4. Preparation of the mRNA Biosensor

In this assay, 15 µL of Cy5-P1 (10 µM), 80 µL of ROX-P2 (10 µM), 100 µL of FAM-P3 (10 µM) were mixed with 650 µL of HEPES buffer (25 mM, 100 mM NaCl, PH 7.4), followed by the addition of 8 µL of GO aqueous solution (1 mg/mL) and reacted at room temperature for 1 min. After that, the fluorescence measurements were carried out on a FL6500 fluorescence spectrometer (Perkin Elmer, Waltham, MA, USA). In the spectral scan mode, the excitation wavelength of Cy5-P1 was 643 nm, and emission was monitored from 650 to 750 nm; for ROX-P2 these were 575 nm and 580–640 nm; FAM-P3 used 494 nm and 520–550 nm. The mixture of three PNA probes (Cy5-P1, ROX-P2, and FAM-P3) and GO nanocomplex was prepared as a PAN-GO-based mRNA biosensor.

### 2.5. Target mRNA Detection In Vitro

#### 2.5.1. The Feasibility of the mRNA Biosensor

The hybrid nano-complex solution (PNA-GO-based mRNA biosensor) was mixed with chemically synthesized AlinOBP14-T1, AlinOBP14-T2, and AlinOBP14-T3, respectively, followed by addition of various concentrations (0–1000 nM) of target mRNA and reacted at room temperature. Then, fluorescence measurements were carried out using a FL6500 fluorescence spectrometer (Perkin Elmer, Waltham, MA, USA) to monitor the hybridization process. For the operation in the spectral scan mode, the excitation and emission wavelengths of three PNA probes were the same as those in the preparation of the mRNA biosensor. 

#### 2.5.2. The Specificity of the mRNA Biosensor

A mixture of hybrid nano-complex (PNA-GO-based mRNA biosensor) was added to solutions consisting of different combinations of three targets (AlinOBP14-T1, AlinOBP14-T2, AlinOBP14-T3, AlinOBP14-T1+AlinOBP14-T2, AlinOBP14-T2+AlinOBP14-T3, AlinOBP14-T1+AlinOBP14-T3, AlinOBP14-T1+AlinOBP14-T2+AlinOBP14-T3), respectively. All of the concentrations of the targets were 1000 nM. The mixtures were reacted at room temperature for 12 h. As a control, the PNA-GO-based mRNA biosensor was added with chemically synthesized mRNA with a random sequence (1000 nM) and reacted at room temperature for 12 h. Then, fluorescence intensities corresponding to FAM, ROX, and CY5 were measured using a FL6500 fluorescence spectrometer (Perkin Elmer, Waltham, MA, USA). In the spectral scan mode, the excitation and emission wavelengths of the PNA probes were same as those used in the preparation of the mRNA biosensor.

### 2.6. Target mRNA Detection in A. Lineolatus

#### 2.6.1. PNA-mRNA Hybridization

The PAN-GO nano-complex solution (100 nL) was injected at the conjunction between the prothorax and mesothorax of each *A. lineolatus* adult using a PLI-100 pico-injector (Harvard Apparatus, Holliston, MA, USA) manipulated by a MP-255 micromanipulator (Sutter, Novato, CA, USA) under a microscope. Each treatment was applied to 10 *A. lineolatus* adults (females or males), and non-injected *A. lineolatus* adults (females or males) were used as a control.

#### 2.6.2. Fluorescence Signals Observation

After 12 h, the expression of the *AlinOBP14* gene in antennae, stylets, the heads without antennae and stylets, thoraces, abdomens, legs, and wings from injected and non-injected *A. lineolatus* adults of both genders was observed by a Zeiss LSM 880 confocal microscope. In brief, the different tissues of injected and non-injected *A. lineolatus* were embedded in Tissue-Tek optimal cutting temperature (O.C.T.) compound (Sakura Finetek, Torrance, CA, USA) and cut into 12 µm slices at −20 °C by using a freezing microtome (Thermo scientific, Cryostar NX50, Waltham, MA, USA). Sections were collected on superfrost plus microscope slides (Fisher Scientific, USA). The slides were dried at room temperature and fixed in 4% paraformaldehyde solution at 4 °C for 30 min, incubated in 0.2 M HCl for 10 min, and washed in PBS buffer. The slides were mounted using ProLong^TM^ Glass antifade mountant (P36980, Thermo Fisher Scientific, Waltham, MA, USA). The tissues were observed using a Zeiss LSM880 confocal microscope (Carl Zeiss Microscopy GmbH, Jena, Germany). Images and colocation characteristics of enhanced fluorescent signals were processed with ZEN 3.2 (Carl Zeiss Microscopy GmbH, Jena, Germany).

#### 2.6.3. Tissue Clearing of A. Lineolatus

In order to observe the sensilla on the epidermis of the vertex and gena and the structure of antennal lobes, the whole body of non-injected *A. lineolatus* adults were cleared following the PEGASOS recirculation procedure [23]. The main steps were as follows: *A. lineolatus* adults were anesthetized and immersed in 4% paraformaldehyde (PFA) at room temperature for 12 h before proceeding to tissue clearing; the adults were then immersed in 20% EDTA (PH 7.0) at 37 °C in a shaker for four days (decalcification). The samples were washed with H_2_O for at least 30 min to elute excessive EDTA. Following this, samples were decolorized with the quadrol decolorization solution for two days and placed in 5% ammonium solution at 37 °C in a shaker. Samples were placed in gradient tB delipidation solutions for 1–2 days and then in tB-PEG for two days for dehydration. Samples were then immersed in the BB-PEG medium at 37 °C for at least one day for clearing.

## 3. Results

### 3.1. Characterization of GO

The chemically synthesized GO was water dispersible. We first characterized the material as prepared GO (1 mg/mL) sheets to confirm their exfoliation using Fourier transform infrared (FT-IR), Raman, X-ray diffraction, and Atomic force microscopy (AFM). The analyses revealed that the average thickness of GO was ~1 nm, characteristic of a fully exfoliated GO sheet. All of the analysis data indicated the successful preparation of GO (Supporting information Figure S3).

### 3.2. Preparation of the mRNA Biosensor

To prepare the PNA-GO-based mRNA biosensor, we studied the adsorption properties of PNA probes on the GO surface and monitored the quenching kinetics by measuring the fluorescence changes of the dye-labeled PNA probes. Then, we optimized the relative amounts of the dye-labeled PNA probes and GO to give more than 97% quenching of the fluorescence by measuring fluorescent spectra of the PNA probes in the presence of GO. The results showed that the addition of 8 µL of GO (1 mg/mL) resulted in 97% fluorescent quenching of 15 µL of CY5-P1 (10 µM), 80 µL of ROX-P2 (10 µM), and 100 µL of FAM-P3 (10 µM) within 1 min (Supporting Information Figure S4). This ratio between the dye-labeled PNA probes and GO was used to detect target mRNA both in vitro and in *A. lineolatus*. The mixture of three PNA probes (P1, P2, and P3) and GO nano-complex was prepared as a PAN-GO-based mRNA biosensor.

### 3.3. Target mRNA Detection In Vitro

**Figure 1:**
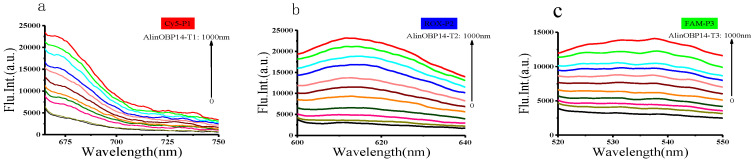
The PNA-GO-based nanocomplex solution (mRNA biosensor) was mixed with chemically synthesized targets of various concentrations (0–1000 nM). The fluorescence signals intensified as the concentration of added targets increased. (**a**) Target mRNA: AlinOBP14-T1. The excitation wavelength was 643 nm. (**b**) Target mRNA: AlinOBP14-T2. The excitation wavelength was 575 nm. (**c**) Target mRNA: AlinOBP14-T3. The excitation wavelength was 494 nm.

In this study, mRNA was detected based on the fluorescence change before and after PNA-RNA hybridization in the solution of GO. Previous studies have shown that GO can effectively protect RNA probes from enzymatic digestion [24]. Moreover, PNA is a DNA mimic having a pseudo-peptide backbone that makes it extremely stable in biological fluids [25]. Initially, the fluorescence dye-labeled PNA probes are absorbed onto the GO surface by π–π stacking interactions accompanied by the quenching of dye fluorescence. Subsequently, the quenched fluorescence signals are recovered, while the dye-labeled PNA probes are hybridized with corresponding complementary target mRNA and desorbed from the GO surface [26]. To evaluate the feasibility of the PAN-GO-based mRNA biosensor, we chose three regions of mRNA (*AlinOBP14*) as the targets, the PAN-GO-based mRNA biosensor was mixed with chemically synthesized AlinOBP14-T1, AlinOBP14-T2, and AlinOBP14-T3 at various concentrations (0–1000 nM), respectively. The results showed that the fluorescent intensity gradually increased within a range of 0–1000 nM of target mRNA (Figure 1), indicating that the PNA probes were adsorbed onto GO and stably hybridized with chemically synthesized target mRNA. 

**Figure 2:**
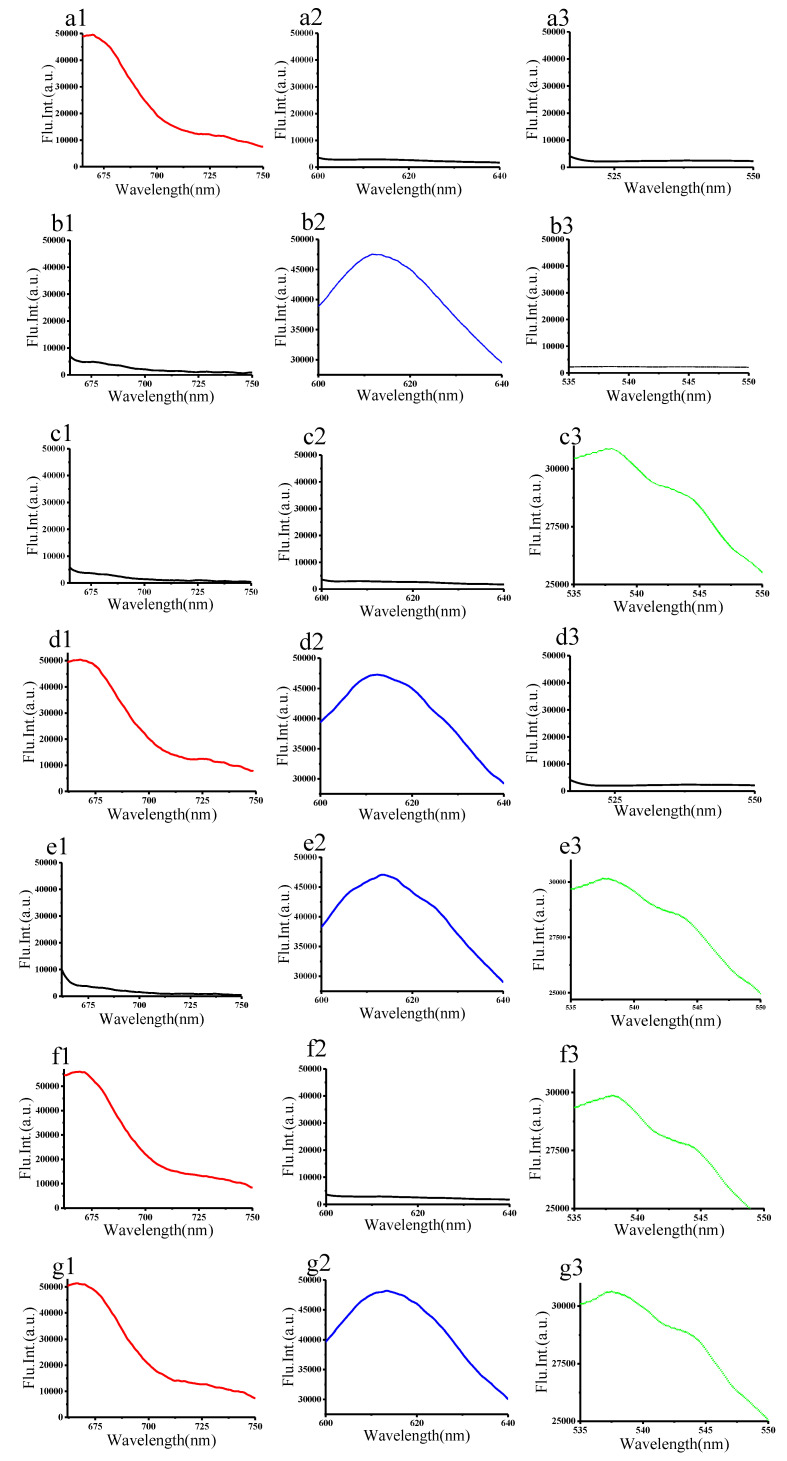
The mRNA biosensor was mixed with solutions consisting of different combinations of the three targets. (**a1**–**a3**) Target mRNA: AlinOBP14-T1. The excitation wavelengths were 643 nm (**a1**), 575 nm (**a2**), and 494 nm (**a3**), respectively. (**b1**–**b3**) Target mRNA: AlinOBP14-T2. The excitation wavelengths were 643 nm (**b1**), 575 nm (**b2**), and 494 nm, (**b3**), separately. (**c1**–**c3**) Target mRNA: AlinOBP14-T3. The excitation wavelengths were 643 nm (**c1**), 575 nm (**c2**), and 494 nm (**c3**), respectively. (**d1**–**d3**) Target mRNA: AlinOBP14-T1 and AlinOBP14-T2. The excitation wavelengths were 643 nm (**d1**), 575 nm (**d2**), and 494 nm (**d3**), separately. (**e1**–**e3**) Target mRNA: AlinOBP14-T2 and AlinOBP14-T3. The excitation wavelengths were 643 nm (**e1**), 575 nm (**e2**), and 494 nm (**e3**), respectively. (**f1**–**f3**) Target mRNA: AlinOBP14-T1 and AlinOBP14-T3. The excitation wavelengths were 643 nm (**f1**), 575 nm (**f2**), and 494 nm (**f3**), separately. (**g1**–**g3**) Target mRNA: AlinOBP14-T1, AlinOBP14-T2 and AlinOBP14-T3. The excitation wavelengths were 643 nm (**g1**), 575 nm (**g2**), and 494 nm (**g3**), respectively.

Often, PNA probes are small-sized (~40 bases). However, the full-length mRNA sequence of *AlinOBP14* is 417bp (GenBank: GQ477035.1). Thus, it was necessary to design multiple probes to simultaneously monitor different regions of the target mRNA. In this study, we designed three PNA probes conjugated with three different fluorescent dyes complementary to the selected regions of target mRNA (probes: CY5-P1, ROX-P2, and FAM-P3; respective target mRNA: AlinOBP14-T1, AlinOBP14-T2, and AlinOBP14-T3). Then, we evaluated the sequence specificity and multiplexed sensing capability of the PNA-GO-based mRNA biosensor in a homogeneous solution. The mRNA biosensor was treated with solutions consisting of different combinations of the three targets. The fluorescence intensity corresponding to Cy5-P1 was increased when mRNA biosensor was mixed with chemically synthesized AlinOBP14-T1, indicating that the PNA probe conjugated with Cy5 hybridized with AlinOBP14-T1 (Figure 2a1–a3). The fluorescence signal of ROX-P2 was intensified (blue) when mRNA biosensor was mixed with chemically synthesized AlinOBP14-T2, indicating that the PNA probe conjugated with ROX hybridized with AlinOBP14-T2 (Figure 2b1–b3). The fluorescence signal of FAM-P3 was intensified (green) when mRNA biosensor was mixed with chemically synthesized AlinOBP14-T3, indicating that the PNA probe conjugated with FAM hybridized with AlinOBP14-T3 (Figure 2c1–c3). The fluorescence intensities corresponding to Cy5-P1 and ROX-P2 were increased (red and blue) when mRNA biosensor was mixed with chemically synthesized AlinOBP14-T1 and AlinOBP14-T2, indicating that PNA probes conjugated with Cy5 and ROX hybridized with AlinOBP14-T1 and AlinOBP14-T2, respectively (Figure 2d1–d3). The fluorescence intensities corresponding to ROX-P2 and FAM-P3 were increased (blue and green) when mRNA biosensor was mixed with chemically synthesized AlinOBP14-T2 and AlinOBP14-T3, indicating that the PNA probes conjugated with ROX and FAM hybridized with AlinOBP14-T2 and AlinOBP14-T3, respectively (Figure 2e1–e3). The fluorescence intensities corresponding to Cy5-P1 and FAM-P3 were increased (red and green) when mRNA biosensor was mixed with chemically synthesized AlinOBP14-T1 and AlinOBP14-T3, indicating that the PNA probes conjugated with Cy5 and FAM hybridized with AlinOBP14-T1 and AlinOBP14-T3, respectively (Figure 2f1–f3). The fluorescence intensities corresponding to Cy5-P1, ROX-P2, and FAM-P3 were increased (red, blue, and green) when mRNA biosensor was mixed with chemically synthesized AlinOBP14-T1, AlinOBP14-T2, and AlinOBP14-T3, indicating that the PNA probes conjugated with Cy5, ROX, and FAM hybridized with AlinOBP14-T1, AlinOBP14-T2, and AlinOBP14-T3, respectively (Figure 2g1–g3). Collectively, fluorescence emission spectra corresponding to Cy5, ROX, and FAM obtained from the study indicated that the three PNA probes hybridized only with complementary targets in a sequence-specific manner and simultaneously sensed multiple targets in homogeneous solutions without cross-reactivity. As a control, exposure of the PNA-GO-based mRNA biosensor to chemically synthesized mRNA with scrambled sequence gave no notable changes in fluorescence intensity (Supporting information Figure S5).

### 3.4. mRNA Detection in A. Lineolatus

Due to the complex intracellular environment, it is not trivial to simultaneously detect multiple targets in insects, and therefore none of the previously reported mRNA biosensors have been employed for multiplexed targets sensing in insects. We examined whether the PAN-GO-based mRNA biosensor could detect the three selected regions of the target mRNA (AlinOBP14-T1, AlinOBP14-T2, and AlinOBP14-T3) in *A. lineolatus*. The PAN-GO nano-complex solution (100 nL) was injected into *A. lineolatus* adults. Non-injected *A. lineolatus* adults were used as a control. After 12 h, the different tissues of *A. lineolatus* adults were cut into 12 µm slices. Fluorescent images of the cells in *A. lineolatus* were obtained using a Zeiss LSM 880 confocal microscope.

**Figure 3:**
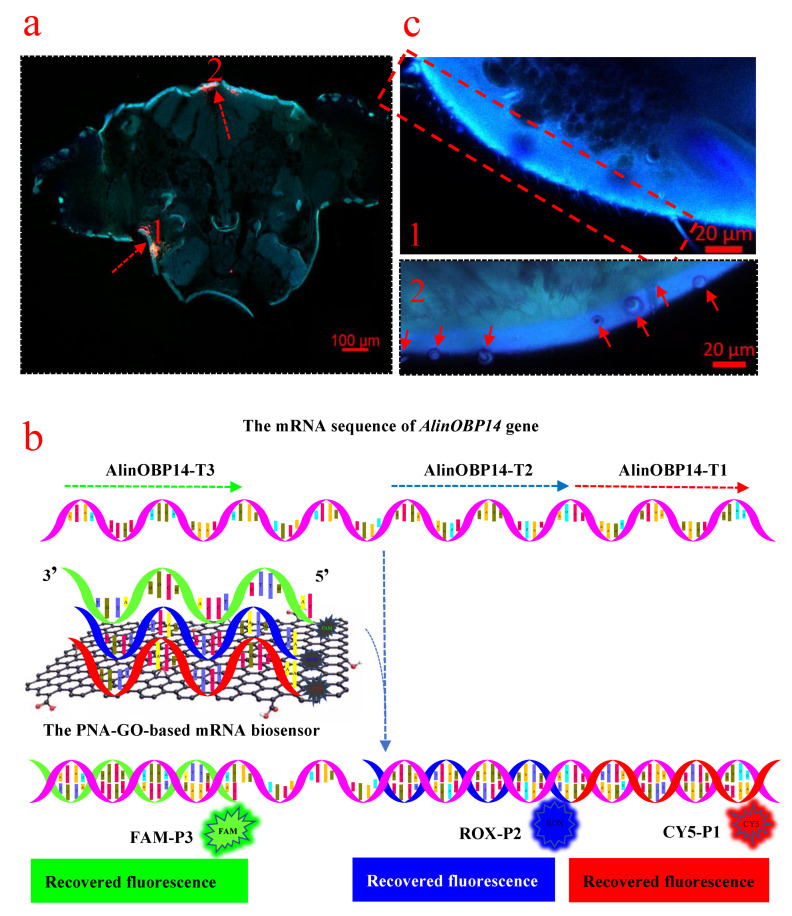
Simultaneous detection of three regions of target mRNA in *A. lineolatus* (probes: CY5-P1, ROX-P2, and FAM-P3; target mRNA: AlinOBP14-T1, AlinOBP14-T2, and AlinOBP14-T3). (**a**) The merged fluorescent image of three different fluorescence channels under the bright-field background showed that the fluorescence signals were intensified beneath the epidermis of the gena (the red arrow marked 1) and vertex (the red arrow marked 2) in the head of *A. lineolatus* (the three fluorescence dyes in the three channels were excited at 643 nm, 575 nm, 488 nm). (**b**) Schematic illustration of the PNA-GO-based mRNA biosensor. The fluorescence signals are recovered when the fluorescent dye-labeled probes initially adsorbed onto the surface of GO detached from GO and hybridized with complementary target mRNA. (**c**) The sensilla distributed at the epidermis of the gena (the red arrow marked 1) and vertex (the red arrow marked 2).

Similar results were obtained from *A. lineolatus* adults (females and males). The female adults were used in the following study unless otherwise indicated. Images of the cells in *A. lineolatus* showed that the intracellularly delivered PAN-GO-based mRNA biosensor induced an increase of the fluorescence intensity beneath the epidermis of the vertex and gena in the head of adult *A. lineolatus* (Figure 3a). This suggested that the three PNA probes hybridized with corresponding complementary target mRNA in *A. lineolatus* (Figure 3b). Non-injected *A. lineolatus* adults were used as a control. Several sensilla were observed at the epidermis of the vertex and gena in the head of adult *A. lineolatus* (Figure 3c). Therefore, we speculated that the *AlinOBP14* gene might be expressed beneath the epidermis of the vertex and gena in the head of adult *A. lineolatus.* To verify this hypothesis, the enhanced fluorescence signals were used for colocalization analysis. From the physical point of view, colocalization usually means that colors emitted by two types of fluorescent molecules occupy the same pixel in the image. In the context of digital imaging, it can be described as the spatial overlap of two dyes in a multichannel image. The possibility of using triple quantification of confocal images with colocalization is susceptible to the problems of sample preparation, microscope setup, and image acquisition and handing [27]. Thus, the colocalization of CY5-T1 and ROX-T2, ROX-T2 and FAM-T3, and Cy5-T1 and FAM-T3 were analyzed.

**Figure 4:**
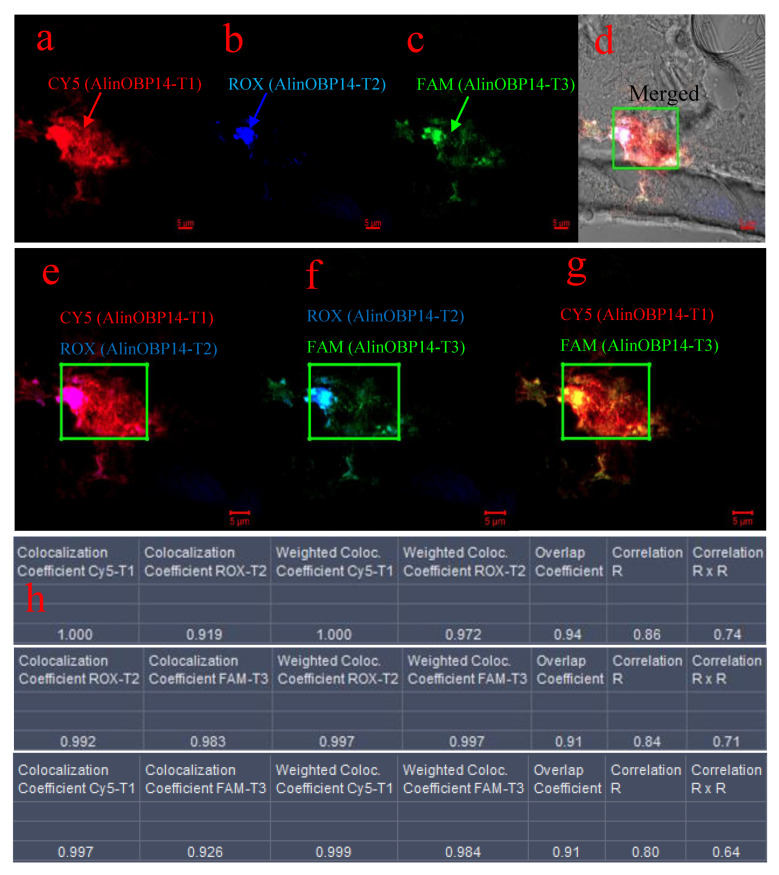
Colocalization analysis of the enhanced fluorescence signals beneath the epidermis of the gena in the head of adult *A. lineolatus.* (**a**) Excitation at 643 nm corresponding to CY5-P1 (red arrow). (**b**) Excitation at 575 nm corresponding to ROX-P2 (blue arrow). (**c**) Excitation at 488 nm corresponding to FAM-P3 (green arrow). (**d**) Merged image of three different fluorescence channels under a bright-field background. The green box indicates fluorescence signals that were intensified beneath the epidermis of the gena. After background correction, images were processed for colocalization analysis. (**e**) The spatial overlap of CY5-P1 and ROX-P2 fluorescence channels in a multichannel image. The green box indicates the area with colocalization of CY5-P1 with ROX-P2. (**f**) The spatial overlap of ROX-P2 and FAM-P3 fluorescence channels in a multichannel image. The green box indicates the area with colocalization of ROX-P2 with FAM-P3. (**g**) The spatial overlap of CY5-P1 and FAM-P3 fluorescence channels in a multichannel image. The green box indicates the area with colocalization of CY5-P1 with FAM-P3. (**h**) The values of coefficients calculated on images for CY5-P1 and ROX-P2, ROX-P2 and FAM-P3, and CY5-P1 and FAM-P3. The scale bar indicates 5 µm (**a**–**g**).

First, we analyzed the enhanced fluorescence signals beneath the epidermis of the gena (Figure 3a, the red arrow marked 1). As shown in Figure 4, the red fluorescence corresponding to CY5-P1 was intensified when excited at 643 nm (Figure 4a), indicating that the GO protected CY5-P1 probe hybridized with AlinOBP14-T1. The blue fluorescence signal corresponding to ROX-P2 was visualized when excited at 575 nm (Figure 4b), demonstrating that the GO protected ROX-P2 probe detached from the GO surface and hybridized with AlinOBP14-T2. The green fluorescence signal corresponding to FAM-P3 was recovered when excited at 488 nm (Figure 4c), suggesting that FAM-P3 initially adsorbed onto the surface of GO detached from GO and formed a double helix with AlinOBP14-T3. Merged images of three different fluorescence channels under the bright-field background showed that fluorescence signals were intensified beneath the epidermis of the gena (Figure 4d). Moreover, to determine the locations of the areas with colocalization, it is equally important to calculate colocalization coefficients. If the coefficients are calculated for a specific area, the results are usually more precise. Thus, coefficients were calculated by selecting areas with colocalization of CY5-T1 and ROX-T2 (Figure 4e), ROX-T2 and FAM-T3 (Figure 4f), and Cy5-T1 and FAM-T3 (Figure 4g). After background correction, colocalization coefficients for Cy5-T1 and ROX-T2 of 1 and 0.919 for the red and blue pairs implied that all red pixels colocalized with blue, and 91.9% of blue pixels colocalized with red. Colocalization coefficients for ROX-T2 and FAM-T3 of 0.992 and 0.983 for blue and green pairs implied that 99.2% of blue pixels colocalized with green, and 98.3% of green pixels colocalized with blue. Colocalization coefficients for Cy5-T1 and FAM-T3 of 0.997 and 0.926 for red and green pairs implied that 99.7% of red pixels colocalized with green, and 92.6% of green pixels colocalized with red. Weighted colocalization coefficients for Cy5-T1 and ROX-T2 of 1 and 0.972 for red and blue pairs implied that all red pixels colocalized with blue, and 97.2% of blue pixels colocalized with red. Weighted colocalization coefficients for ROX-T2 and FAM-T3 of 0.997 and 0.997 for blue and green pairs implied that 99.7% blue pixels colocalized with green, and 99.7% of green pixels colocalized with blue. Weighted colocalization coefficients for Cy5-T1 and FAM-T3 of 0.999 and 0.984 for red and green pairs implied that 99.9% red pixels colocalized with green, and 98.4% of green pixels colocalized with red. Calculations on images showed that MOC values (Overlap coefficients according to Manders) were 0.94 for a Cy5-T1-ROX-T2 pair, 0.91 for a ROX-T2-FAM-T3 pair, and 0.91 for a Cy5-T1-FAM-T3 pair, indicating that 94% of Cy5-T1 channel and ROX-T2 channel colocalized, 91% of ROX-T2 channel and FAM-T3 channel colocalized, and 91% of Cy5-T1 channel and FAM-T3 channel colocalized. Pearson’s correlation coefficient (Correlation R) values were 0.86 for a Cy5-T1-ROX-T2 pair, 0.84 for a ROX-T2-FAM-T3 pair, and 0.80 for a Cy5-T1-FAM-T3 pair, indicating that 86% correlation of the intensity distribution between Cy5-T1 channel and ROX-T2 channel, 84% correlation of the intensity distribution between ROX-T2 channel and FAM-T3 channel, and 80% correlation of the intensity distribution between Cy5-T1 channel and FAM-T3 channel. Correlation (R × R) values were 0.74 for a Cy5-T1-ROX-T2 pair, 0.71 for a ROX-T2-FAM-T3 pair, 0.64 for a Cy5-T1-FAM-T3 pair, indicating that 74% correlation of Cy5-T1 channel and ROX-T2 channel, 71% correlation of ROX-T2 channel and FAM-T3 channel, 64% correlation of Cy5-T1 channel and FAM-T3 channel (Figure 4h). Collectively, all of the data indicated that CY5-P1, ROX-P2, and FAM-P3 were located beneath the epidermis of the gena. This suggests that the three PNA probes hybridized with corresponding complementary target mRNA beneath the epidermis of the gena, and *AlinOBP14* is expressed beneath the epidermis of the gena in the head of *A. lineolatus*.

**Figure 5:**
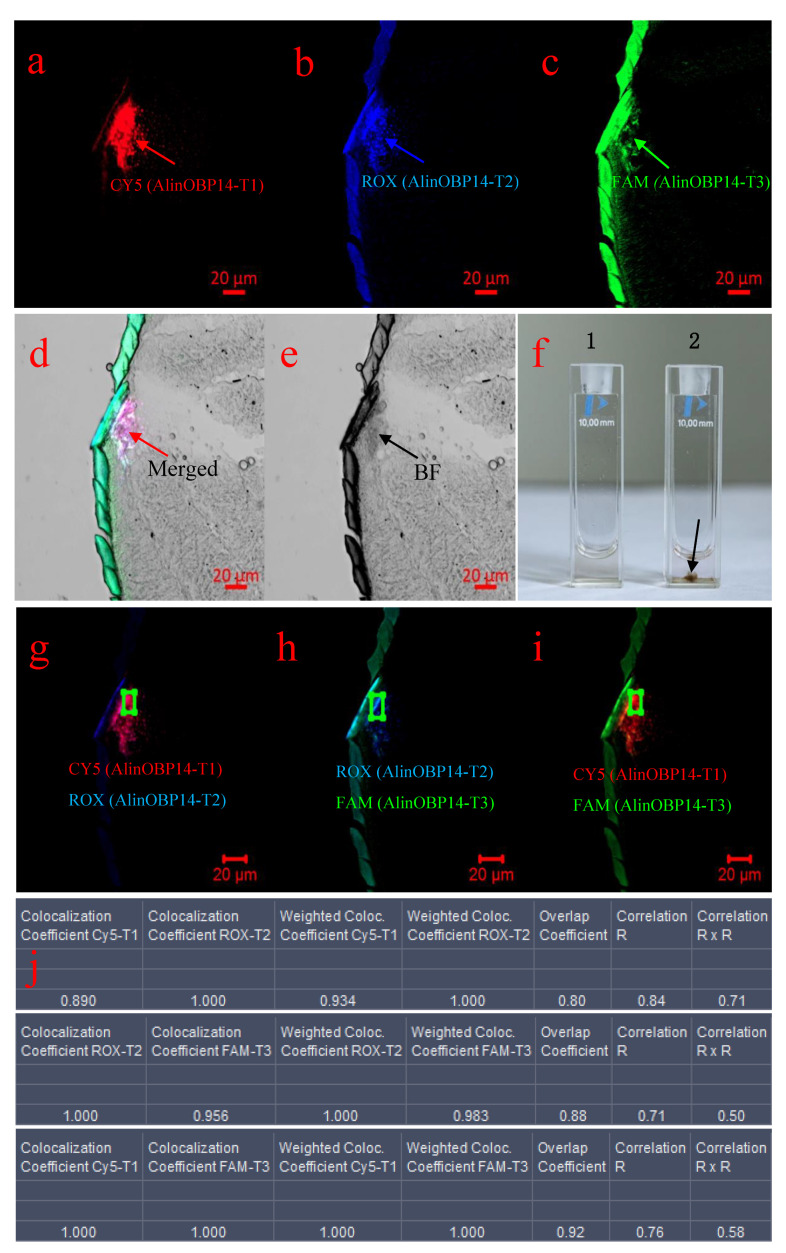
Colocalization analysis of the enhanced fluorescence signals beneath the epidermis of the vertex in the head of adult *A. lineolatus.* (**a**) Excitation at 643 nm corresponding to CY5-P1 (red arrow). (**b**) Excitation at 575 nm corresponding to ROX-P2 (blue arrow). (**c**) Excitation at 488nm corresponding to FAM-P3 (green arrow). (**d**) Merged image of three different fluorescence channels under a bright-field background showing that the fluorescence signals were intensified beneath the epidermis of the vertex (red arrow). (**e**) A bright-field image of the epidermis of the vertex (black arrow). (**f**) 1 indicates that the solution of PNA-GO-based mRNA biosensor. 2 indicates that the solution of PNA-GO-based mRNA biosensor was mixed with chemically synthesized targets (AlinOBP-T1, AlinOBP-T2, and AlinOBP-T3) in vitro. The black arrow points to the aggregation of GO sheets in vitro. After background correction, images were processed for colocalization analysis. (**g**) The spatial overlap of CY5-P1 and ROX-P2 fluorescence channels in a multichannel image. The green box indicates the area with colocalization of CY5-P1 with ROX-P2. (**h**) The spatial overlap of ROX-P2 and FAM-P3 fluorescence channels in a multichannel image. The green box indicates the area with colocalization of ROX-P2 with FAM-P3. (**i**) The spatial overlap of CY5-P1 and FAM-P3 fluorescence channels in a multichannel image. The green box indicates the area with colocalization of CY5-P1 with FAM-P3. (**j**) The values of coefficients calculated using images for CY5-P1 and ROX-P2, ROX-P2 and FAM-P3, and CY5-P1 and FAM-P3, respectively. The scale bar indicates 20 µm (**a**–**e**, **g**–**i**).

We next analyzed the enhanced fluorescence signals beneath the epidermis of the vertex (Figure 3a, red arrow marked 2). As mentioned above, the enhanced fluorescence signals indicated that the three probes initially adsorbed onto the surface of GO detached from GO and formed a double helix with the corresponding complementary targets (Figure 5a–d). Interestingly, we found the intracellularly delivered PAN-GO-based mRNA biosensor not only induced an increase of the fluorescence intensity when the probes hybridize with targets but also induced the aggregation of GO sheets (Figure 5e). We also observed this phenomenon when the PAN-GO-based mRNA biosensor was incubated with chemically synthesized targets in vitro (Figure 5f). Therefore, we speculated that the GO sheets aggregated and formed a stacked structure when the PNA probes detached from GO and formed double helices with the targets. Similarly, coefficients were calculated by selecting areas with colocalization of CY5-T1 and ROX-T2 (Figure 5g), ROX-T2 and FAM-T3 (Figure 5h), and Cy5-T1 and FAM-T3 (Figure 5i). After background correction, colocalization coefficients for Cy5-T1 and ROX-T2 of 0.89 and 1 for red and blue pairs implied that 89% of red pixels colocalized with blue, and all of the blue pixels colocalized with red. Colocalization coefficients for ROX-T2 and FAM-T3 of 1 and 0.956 for blue and green pairs implied that all of the blue pixels colocalized with green, and 95.6% of green pixels colocalized with blue. Colocalization coefficients for Cy5-T1 and FAM-T3 of 1 and 1 for red and green pairs implied that all of the red pixels colocalized with green and all of the green pixels colocalized with red. Weighted colocalization coefficients for Cy5-T1 and ROX-T2 of 0.934 and 1 for red and blue pairs implied that 93.4% of red pixels colocalized with blue, all of the blue pixels colocalized with red. Weighted colocalization coefficients for ROX-T2 and FAM-T3 of 1 and 0.983 for blue and green pairs implied that all of the blue pixels colocalized with green, 98.3% of green pixels colocalized with blue. Weighted colocalization coefficients for Cy5-T1 and FAM-T3 of 1 and 1 for red and green pairs implied that all of the red pixels colocalized with green, and all of the green pixels colocalized with red. Calculations on images showed that MOC values (Overlap coefficient according to Manders) were 0.8 for a Cy5-T1-ROX-T2 pair, 0.88 for a ROX-T2-FAM-T3 pair, 0.92 for a Cy5-T1-FAM-T3 pair, indicating that 80% of Cy5-T1 channel and ROX-T2 channel colocalized, 88% of ROX-T2 channel and FAM-T3 channel colocalized, 92% of Cy5-T1 channel and FAM-T3 channel colocalized. Pearson’s correlation coefficient (Correlation R) values were 0.84 for a Cy5-T1-ROX-T2 pair, 0.71 for a ROX-T2-FAM-T3 pair, and 0.76 for a Cy5-T1-FAM-T3 pair, indicating that 84% correlation of the intensity distribution between Cy5-T1 channel and ROX-T2 channel, 71% correlation of the intensity distribution between ROX-T2 channel and FAM-T3 channel, 76% correlation of the intensity distribution between Cy5-T1 channel and FAM-T3 channel. Correlation (R × R) values were 0.71 for a Cy5-T1-ROX-T2 pair, 0.5 for a ROX-T2-FAM-T3 pair, and 0.58 for a Cy5-T1-FAM-T3 pair, indicating that 71% correlation of Cy5-T1 channel and ROX-T2 channel, 50% correlation of ROX-T2 channel and FAM-T3 channel, 58% correlation of Cy5-T1 channel and FAM-T3 channel (Figure 5j). Collectively, all of the data indicated that CY5-P1, ROX-P2, and FAM-P3 were located beneath the epidermis of the vertex. This suggests that three PNA probes hybridized with corresponding complementary target mRNA beneath the epidermis of vertex, and *AlinOBP14* is expressed beneath the epidermis of the vertex in the head of *A. lineolatus*.

**Figure 6:**
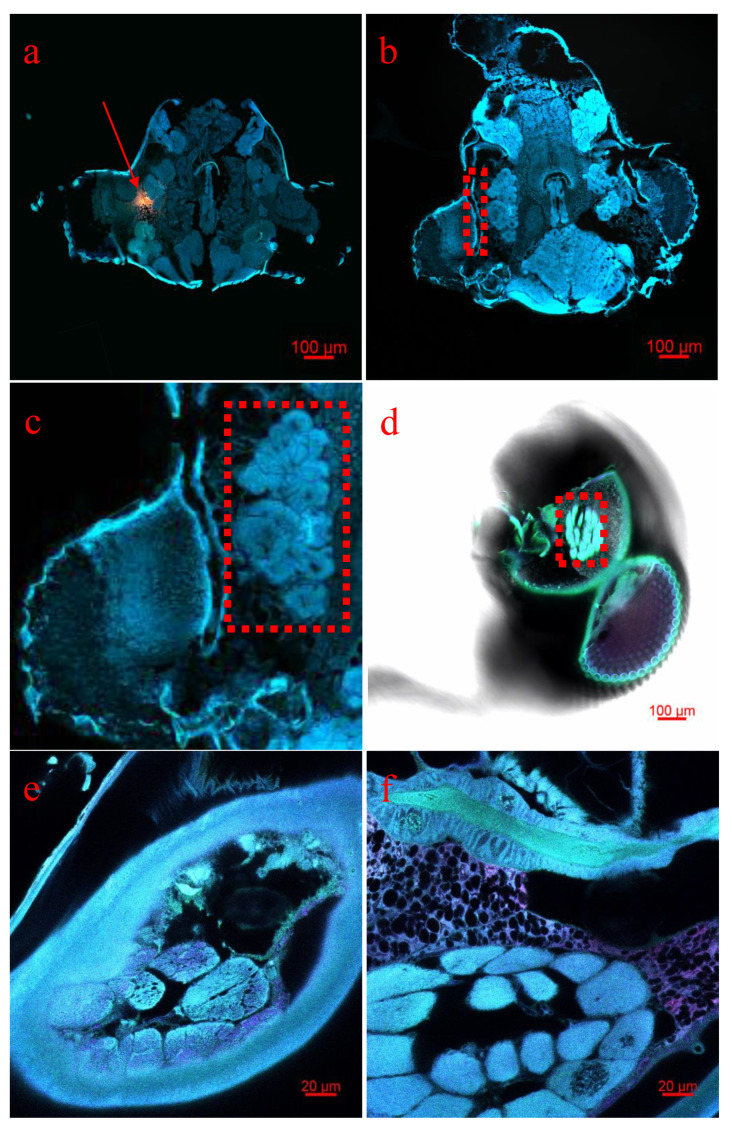
The analysis of the enhanced fluorescence signals in the peripheral antennal lobe. (**a**) The merged fluorescent image of three different fluorescence channels under the bright-field background showed that the fluorescence signals were intensified in the peripheral antenna lobe (The three fluorescence dyes in three channels were excited at 643 nm, 575 nm, 488 nm). The red arrow points to fluorescent signals of PNA probes. The scale bar indicates 100 μm. (**b**) Non-injected *A. lineolatus* adults were used as a control. The red box indicates the peripheral of antennal lobe. The scale bar indicates 100 μm. (**c**) The antennal lobe of *A. lineolatus* in Fig 6b was amplified. (**d**) After the whole body of *A. lineolatus* adults were following PEGASOS recirculation procedure, the antennal lobe in the head of *A. lineolatus* was observed using a Zeiss LSM 880 confocal microscope (10×). The scale bar indicates 100 μm. (**e**–**f**) Different layers of the antennal lobe observed under using a Zeiss LSM 880 confocal microscope (63×). The scale bar indicates 20 μm.

Additionally, fluorescent signals of PNA probes were observed in the peripheral antennal lobe. Thus, we speculated that *AlinOBP14* might also be expressed in the peripheral antennal lobe (Figure 6a). This assumption was further supported by analyzing the structure of the antennal lobe of *A. lineolatus* [28,29]. Non-injected *A. lineolatus* adults were used as a control (Figure 6b–c). Due to the inherent autofluorescence, *A. lineolatus* adults were cleared following the polyethylene glycol-associated solvent system (PEGASOS) recirculation procedure. The antennal lobe in the head of *A. lineolatus* is a sphere-shaped part of the deutocerebrum (Figure 6d), containing glomeruli, spheroidal neuropilar structures (Figure 6e–f). To our surprise, the antennal lobe takes up a relatively large proportion of the entire brain in *A. lineolatus*, indicating that the antenna lobe plays an eminent role in its life.

## 4. Discussion

OBPs are considered to bind and transport hydrophobic compounds across sensillum lymph to the corresponding receptors [30,31]. The ability of OBP proteins to bind and solubilize small hydrophobic compounds can be adapted to a variety of roles in biological systems. In other words, the distinct tissue-biased distribution of OBP genes in insects is related to distinct biological functions [32,33,34,35]. Hence, detection of the expression of OBP genes in insects is central to questions concerning the biological functions of OBP genes. In *A. lineolatus*, about thirty-four putative OBP genes, including classic OBPs and Plus-C OBPs, were identified from antennal transcriptome data [9]. The expression profiles indicated that *AlinOBP1*, *AlinOBP2*, *AlinOBP3*, *AlinOBP4*, *AlinOBP5*, *AlinOBP6*, *AlinOBP8*, *AlinOBP12*, and *AlinOBP13* were mainly expressed in antennae; *AlinOBP9* and *AlinOBP10* were expressed in antennae, head, and legs; *AlinOBP11* was specifically expressed in legs. The Plus-C OBP *AlinOBP7* was mainly expressed in antennae, head, and legs, another Plus-C OBP *AlinOBP14* was highly expressed in the head [21], while *AlinOBP18*, *AlinOBP20*, *AlinOBP21*, *AlinOBP23*, *AlinOBP26*, *AlinOBP27*, *AlinOBP28*, *AlinOBP29*, and *AlinOBP33* were uniquely or primarily expressed in antennae; *AlinOBP22, AlinOBP24, AlinOBP31, AlinOBP32,* and *AlinOBP34* were expressed both in antennae and body parts; *AlinOBP19*, *AlinOBP25*, and *AlinOBP30* were strongly expressed in body parts. The classic OBPs play olfactory or gustatory roles, although the functions of Plus-C OBPs remain largely unknown [9]. Quantitative real-time PCR analysis suggested that the Plus-C OBP *AlinOBP14* was highly expressed in the adult head, and fluorescence-based competitive binding assays showed that β-ionone, nerolidol, *trans*, *trans*-farnesol and insect JHIII strongly bound to *AlinOBP14* [36]. However, the expression profile analysis of *AlinOBP14* with qPCR was limited to in vitro study, and the functional analysis of *AlinOBP14* requires further in vivo evidence. In the present work, we reported a PNA-GO-based mRNA biosensor that allows simple, specific detection of the expression of the *AlinOBP14* gene in *A. lineolatus*. We employed PNA instead of RNA as the capture probes to hybridize with target mRNA, as PNA possesses many advantages [37]:(1) PNA has a uncharged backbone that exhibits tighter binding to GO, which can reduce background fluorescence signal and lower the signal to noise ratio [38]; (2) The neutral amide backbone of PNA typically hybridizes to complementary targets with high affinity and stability [39]; (3) PNA is resistant to nuclease- and protease-mediated degradation, effectively protecting the probes from enzymatic cleavage in living cells [40]; (4) PNA probes are adsorbed onto GO, and thus PNA can be loaded onto GO and delivered through the cell membrane to hybridize with target mRNA in living cells [41]. 

In this study, we found that the intracellularly delivered PAN-GO-based mRNA biosensor induced an increase in the fluorescence intensity beneath the epidermis of the vertex and gena in the head of adult *A. lineolatus*. Moreover, we found several sensilla located at the epidermis of the vertex and gena in *A. lineolatus* adults. Several divergent *Drosophila* OBPs are expressed in olfactory and gustatory sensilla [42]. Generally, the putative function of sensilla can be induced from the number of pores and internal dendrites [43]. It has been proposed that *AlinOBP14* is expressed in the cells beneath the epidermis of the vertex and gena in the head of adult *A. lineolatus*. To verify this hypothesis, the enhanced fluorescence signals beneath the epidermis of the vertex and gena of *A. lineolatus* were used for colocalization analysis. The results indicated that CY5-P1, ROX-P2, and FAM-P3 were located beneath the epidermis of the vertex and gena. Therefore, three PNA probes hybridized with corresponding complementary target mRNA beneath the epidermis of the vertex and gena, and *AlinOBP14* was expressed in the sensilla beneath the epidermis of the vertex and gena of *A. lineolatus*. In sensilla, ligands enter the sensillum lymph to interact with receptor molecules located on the neuronal dendrites, OBPs functions as carriers for binding and transport of ligands, and they protect chemical ligands from enzymatic modification in the sensillum lymph. *AlinOBP14* was detected beneath the epidermis of the vertex and gena. Is *AlinOBP14* related to olfactory recognition? Fluorescence-based competitive binding assays showed that three terpenoids, including β-ionone, nerolidol, and trans, trans-farnesol strongly bind to *AlinOBP14* [36]. Those three terpenoids are plant volatiles [44,45,46]. Therefore, we speculated that *AlinOBP14* functions as a carrier for the three terpenoids.

In addition, *trans*, *trans*-farnesol is not only a plant volatile but also an endogenous compound that functions as a precursor for insect JH biosynthesis [47,48], and fluorescence-based competitive binding assays showed that insect JHIII strongly binds to *AlinOBP14* [36]. Is *AlinOBP14* also related to bind and transport insect JH in *A. lineolatus*? In this study, we also found that the intracellularly delivered PAN-GO-based mRNA biosensor induced an increase of the fluorescence intensity in the peripheral antennal lobe, suggesting that *AlinOBP14* is expressed in the peripheral antennal lobe. To verify this hypothesis, the enhanced fluorescence signals in the peripheral antennal lobe were used for colocalization analysis. CY5-P1, ROX-P2, and FAM-P3 were detected in the peripheral antennal lobe. This suggested that the three PNA probes hybridized with corresponding complementary target mRNA in the peripheral antennal lobe, indicating expression of *AlinOBP14*. What is the connection among *AlinOBP14*, JH, and the antennal lobe? Previous studies have shown that JH can affect honey bee behavioral development by altering levels of the neuromodulators in the antennal lobe [49] and can activate the responsiveness of olfactory interneurons in the antennal lobe [50,51,52]. Thus, we propose that *AlinOBP14* is also expressed in the peripheral antennal lobe of *A. lineolatus* and functions as a carrier for the endogenous compounds, including precursors for JH and JHIII. However, these hypotheses require experimental testing, for example via monitoring the expression level of *AlinOBP14* in the head of *A. lineolatus* after stimulation with these four compounds. Although this method can be used to transduce the complex process of the *AlinOBP14* gene expression into visual images and allows simple, specific detection of the expression of the *AlinOBP14* gene, the quantitative monitoring of mRNA expression level of *AlinOBP14* cannot be performed, as the PNA probes hybridize with target mRNA in *A. lineolatus* at room temperature over a 12 h period, and the expression level of *AlinOBP14* gene will decreased when the PNA probes hybridizes with target mRNA.

In this study, we found that the PNA-GO-based mRNA biosensor enabled monitoring of the expression of the *AlinOBP14* gene in *A. lineolatus*. PNA probes were adsorbed onto GO, and thus could be loaded onto GO and delivered through the cell membrane to hybridize with target mRNA in living cells without nuclease- or protease-mediated degradation. Furthermore, the mRNA biosensor had sufficient selectivity to differentiate complementary and non-complementary targets. Additionally, the PNA probes hybridized only with complementary mRNA in a sequence-specific manner and simultaneously sensed multiple targets in a homogeneous solution without cross-reactivity. However, the quantitative monitoring of mRNA expression levels in insects still needs to be combined with conventional methods, such as Northern blotting and real-time qPCR.

Overall, the present study shows *AlinOBP14* is expressed beneath the epidermis of the vertex and gena and in the peripheral antennal lobe in the head of adult *A. lineolatus*. This study is the first to detect the expression of a Plus-C OBP gene in *A. lineolatus* using the mRNA biosensor. Furthermore, this method could be used to provide a theoretical basis for pest management strategies based on manipulating insects away from food or interrupting their behavior, for example, attractants and repellents could be screened out from the ligands for use in pest control. In addition, we expect that the PNA-GO-based mRNA biosensor can be applicable in olfactory-related research in insects. For example, the detection of the expression of OBP genes in insects and monitoring of the expression of olfactory receptor (OR) genes would facilitate research concerning the molecular basis for olfactory reception of insects. This would in turn aid implementation of the olfactory related genes as pest control targets via interference with pest olfactory recognition [53].

## 5. Conclusions

We designed a PNA-GO-based mRNA biosensor to detect chemically synthesized target mRNA in vitro and the expression of *AlinOBP14* in *A. lineolatus*. *AlinOBP14* was expressed beneath the epidermis of the vertex and gena, and in the peripheral antennal lobe. Our previous work showed that β-ionone, nerolidol, *trans*, *trans*-farnesol, and insect JHIII strongly bind to *AlinOBP14*. Thus, we propose that *AlinOBP14* plays a dual role in the recognition of ligands, not only functioning as a carrier for terpenoids but also as a carrier for endogenous compounds. 

## Supplementary Materials

The following are available online at https://www.mdpi.com/article/10.3390/insects12050422/s1, Figure S1. Schematic illustration of the PNA-GO-based mRNA biosensor to detect chemically synthesized target mRNA in vitro and the expression of *AlinOBP14* gene in *Adelphocoris lineolatus*; Table S1. Sequence information of target mRNA and peptide nucleic acid probes; Figure S2. HPLC profiles and MS spectra of PNA probes; Figure S3. Characterization of GO; Figure S4. Fluorescence spectra of Cy5-, ROX-, FAM-PNAs with or without GO in HEPES buffer (25 mM, 100 mM NaCl, PH 7.4); Figure S5. Fluorescence spectra for PNA probes in the presence of GO (black) and incubation with chemically synthesized mRNA with scrambled sequence (purple).
