# Supplementary material for: OBP14 (Odorant-Binding Protein) Sensing in Adelphocoris lineolatus Based on Peptide Nucleic Acid and Graphene Oxide
Source: Insects. 2021 May 8;12(5):422. doi: 10.3390/insects12050422 (PMC8151863; doi:10.3390/insects12050422)
Supplement: Supplementary file 1 [file insects-12-00422-s001.zip › insects-1156515-supplementary.pdf]

Supplementary Materials:

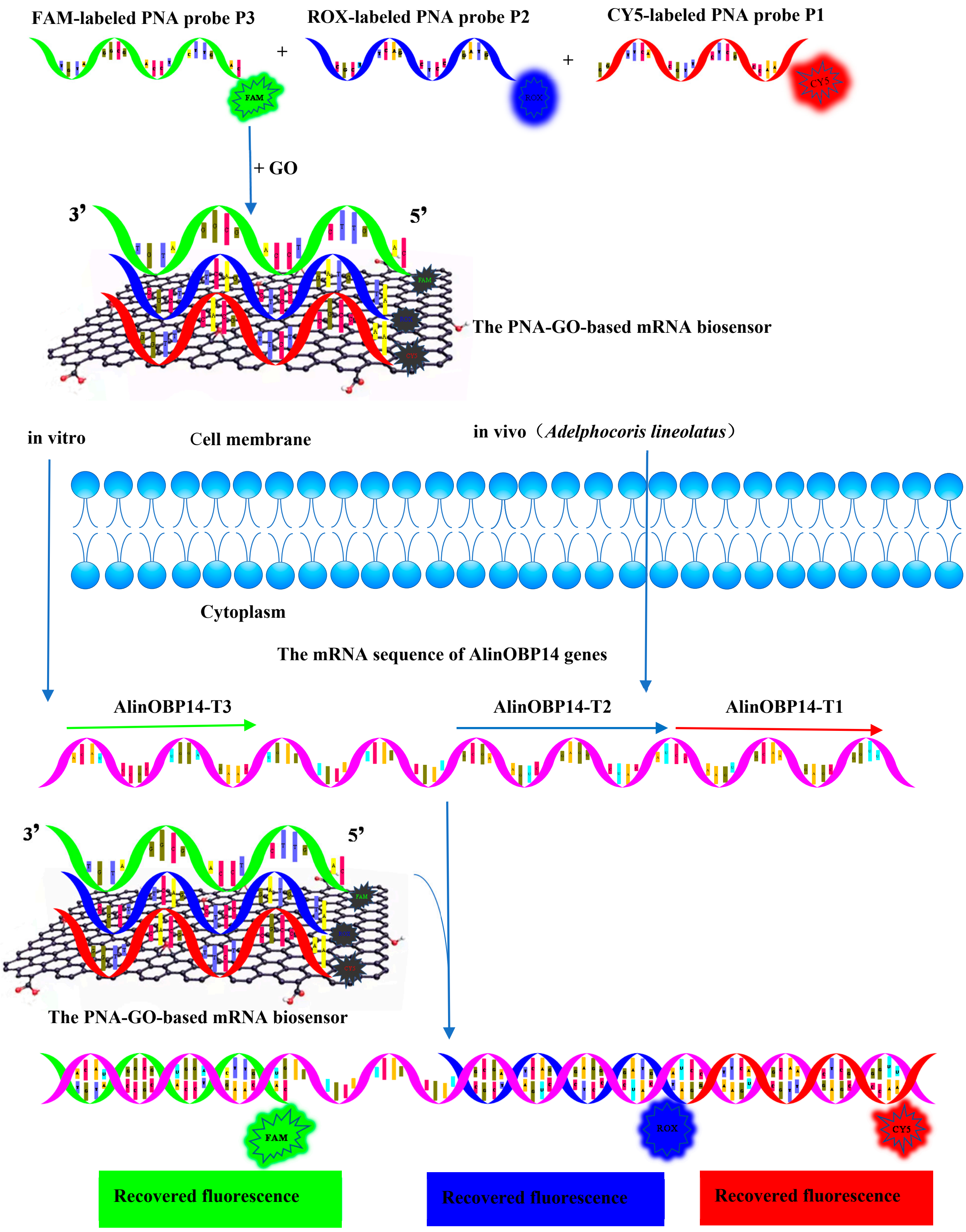

Figure S1. Schematic illustration of the PNA-GO-based mRNA biosensor to detect chemically synthesized target mRNA *in vitro* and the expression of *AlinOBP14* gene in *A. lineolatus*. Three PNA

probes were prepared as conjugated with three different fluorescent dyes complementary for the selected three regions of target mRNA（Probes: CY5-P1, ROX-P2, and FAM-P3, target mRNA: AlinOBP14-T1, AlinOBP14-T2, and AlinOBP14-T3）. The fluorescence signals were quenched when the fluorescence dye-labeled probes initially adsorbed onto the surface of GO surface. Subsequently, the fluorescence signals were recovered when the fluorescence dye-labeled probes detach from GO and hybridize only with corresponding complementary mRNA in a sequence-specific manner both in vitro and in *A. lineolatus.*

Table S1. Sequence information of target mRNA and peptide nucleic acid probes.

species	Target mRNA	mRNA sequence (5′-3′)	PNA-probes (5′-3′)
Adelphocoris lineolatus	AlinOBP14-T1	CCAAGUGCAAGAGCGGUU	P1: CY5-OO-AACCGCTCTTGCACTTGG
	AlinOBP14-T2	GCGAAGUCGAGGCUACAU	P2: ROX-OO-ATGTAGCCTCGACTTCGC
	AlinOBP14-T3	ACAUCCGCUGGAGAACUG	P3: FAM-OO-CAGTTCTCCAGCGGATGT
	random sequence	R: UGAUAAAGCGGUGUUC	

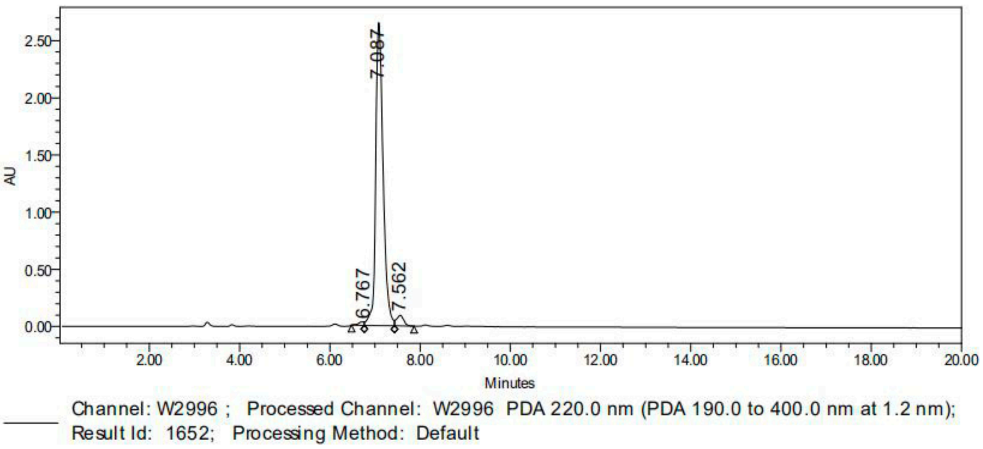

	RT	Area	Height (µV)	Width (sec)	% Area
1	6.767	265518	29072	17.000	0.85
2	7.087	30054638	2655669	40.000	95.85
3	7.562	1034184	92445	26.000	3.30

HPLC

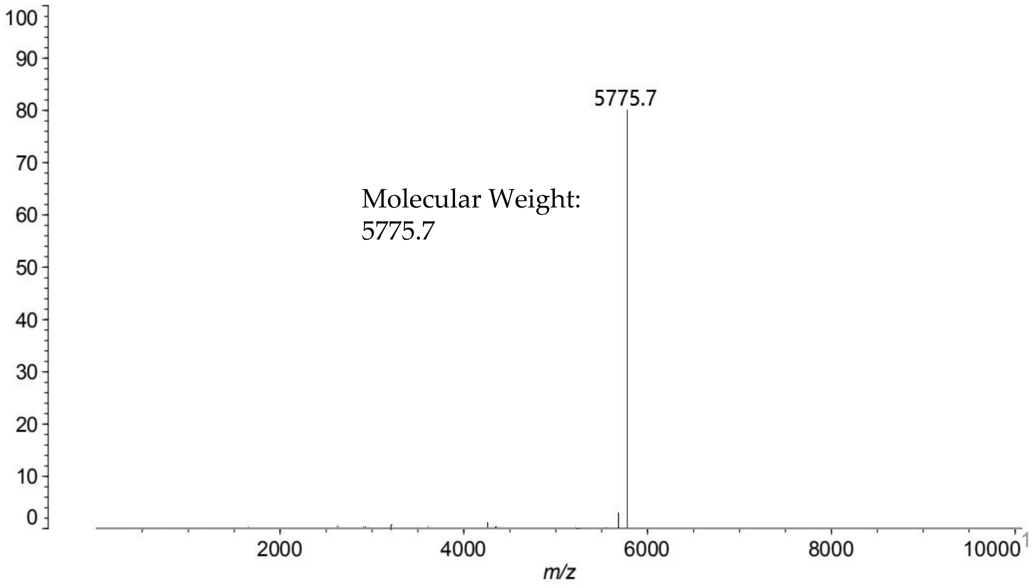

MS

(a)

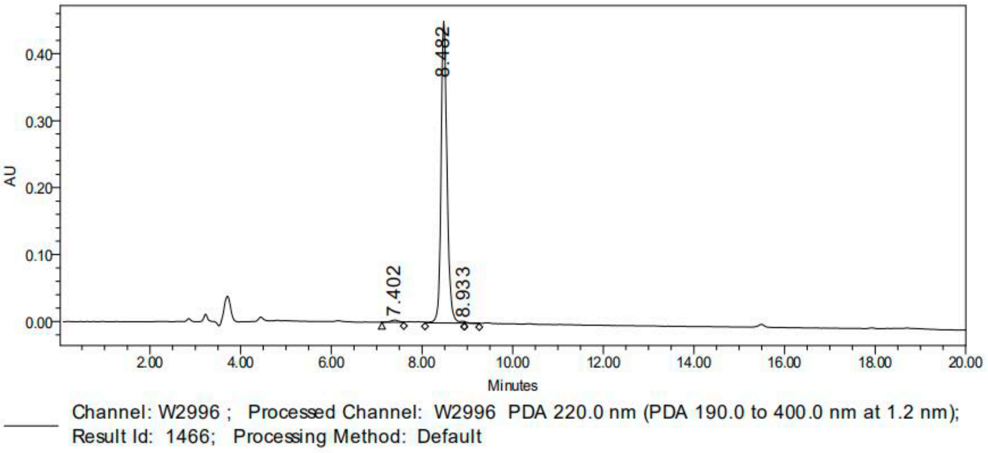

Processed Channel Descr.: W2996 PDA 220.0 nm (PDA 190.0 to 400.0 nm at 1.2 nm)

	Processed Channel Descr.	RT	Area	% Area
1	W2996 PDA 220.0 nm (PDA 190.0 to 400.0 nm at 1.2 nm)	7.402	45169	1.08
2	W2996 PDA 220.0 nm (PDA 190.0 to 400.0 nm at 1.2 nm)	8.482	4119203	98.59
3	W2996 PDA 220.0 nm (PDA 190.0 to 400.0 nm at 1.2 nm)	8.933	13912	0.33

HPLC

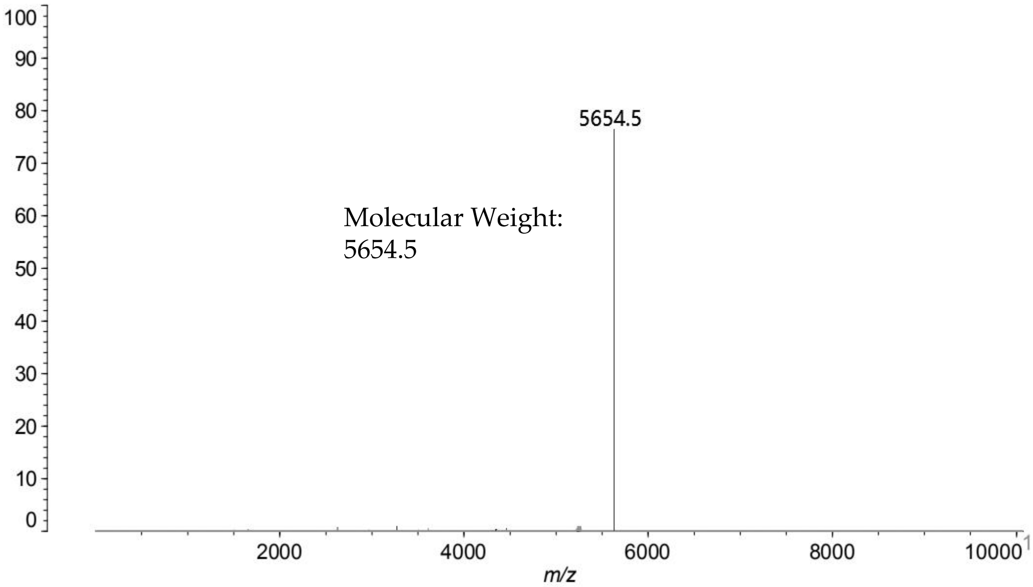

MS

(b)

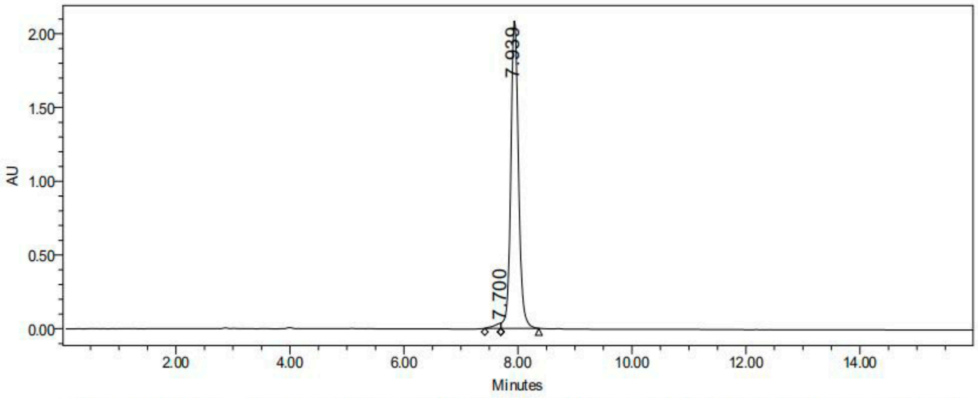

Channel: W2996 ; Processed Channel: W2996 PDA 220.0 nm (PDA 190.0 to 400.0 nm at 1.2 nm);
Result Id: 1468; Processing Method: Default

Processed Channel Descr.: W2996 PDA 220.0 nm (PDA 190.0 to 400.0 nm at 1.2 nm)

	Processed Channel Descr.	RT	Area	% Area
1	W2996 PDA 220.0 nm (PDA 190.0 to 400.0 nm at 1.2 nm)	7.700	281901	1.41
2	W2996 PDA 220.0 nm (PDA 190.0 to 400.0 nm at 1.2 nm)	7.939	19768797	98.59

HPLC

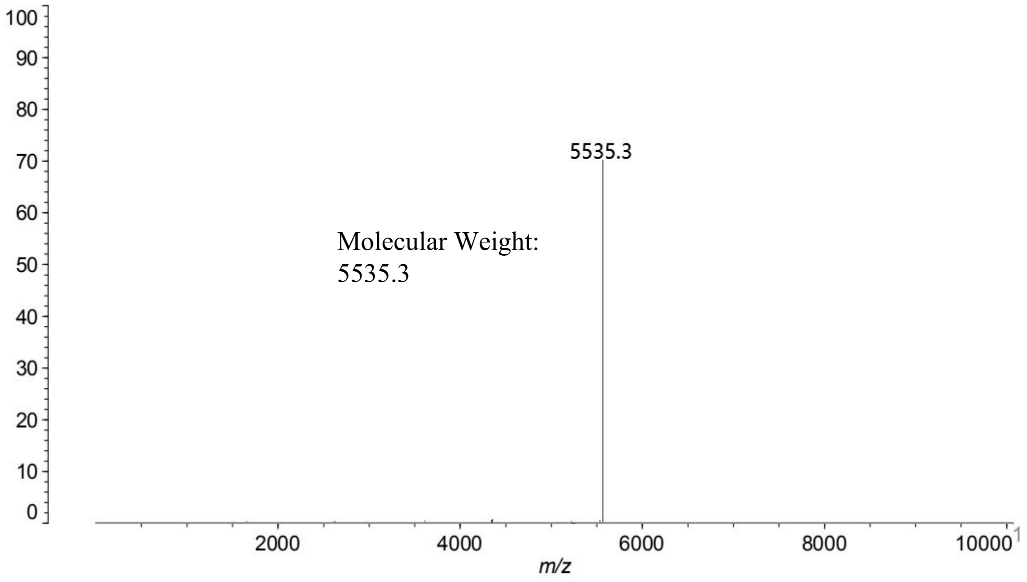

MS

(c)

Figure S2. HPLC profiles and MS spectra of PNA probes. (a) Cy5-P1, (b) ROX-P2, (c) FAM-P3.

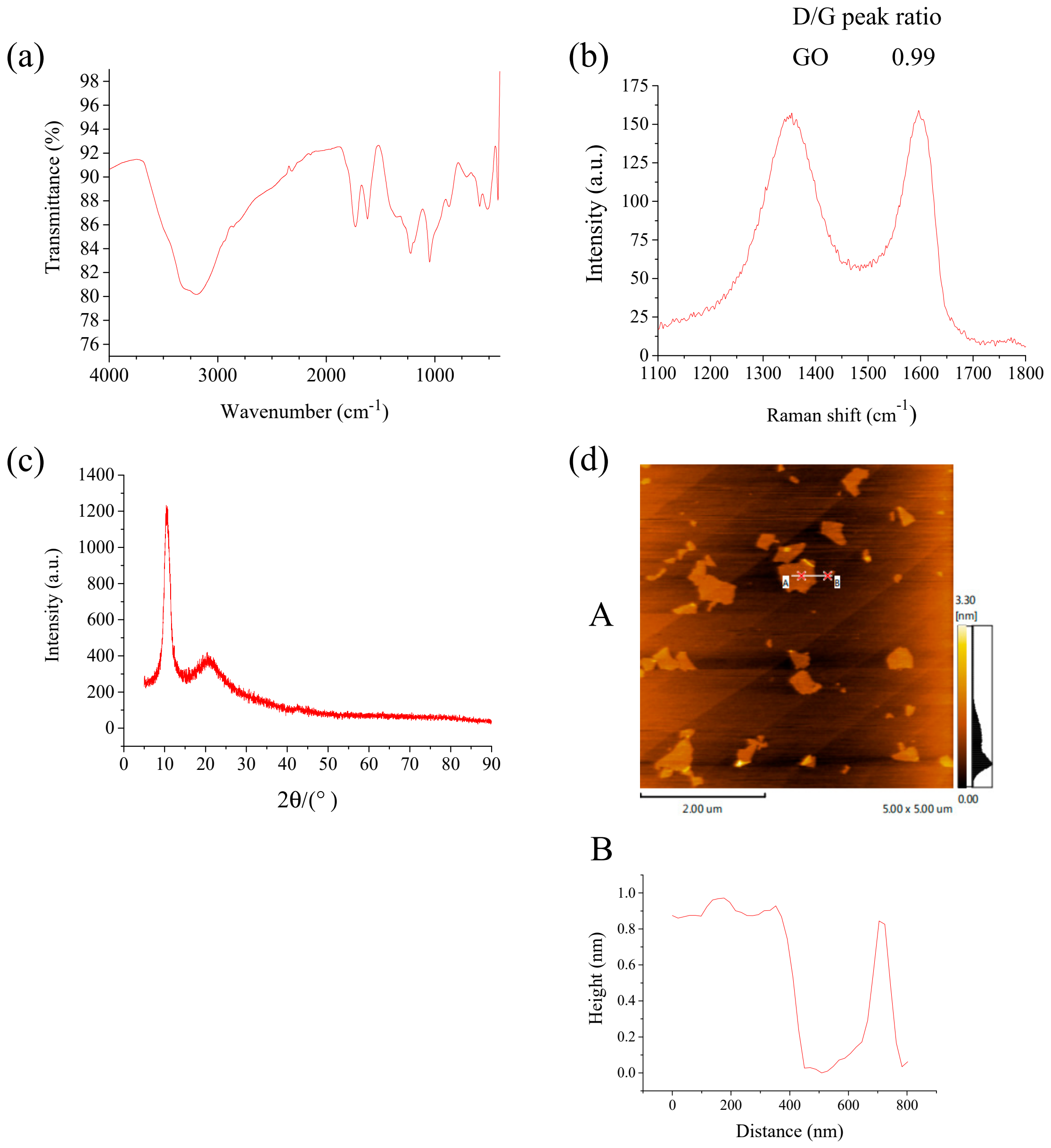

Figure S3. Characterization of GO. (a) Fourier transform infrared (FT-IR) study confirmed the successful oxidation of graphite to graphite oxide which showed several peaks from oxygen-containing functional groups such O-H vibration at 3408 cm^{-1}, C=O stretching at 1731 cm^{-1}, O-H deformation at 1620 cm^{-1}, C-O-C stretching at 1222 cm^{-1} and C-OH stretching at 1048 cm^{-1}. (b) Raman spectrum of GO showed D-band derived from the defected graphitic structures is located at 1350 cm^{-1} and G-band derived from the pristine graphitic structures is located at 1597 cm^{-1}, and the relative intensity ratio of D/G band was 0.99. (c) X-ray diffraction (XRD) spectra of the graphene oxide (GO) showed the diffraction peak for the corresponding (100) reflection is about 2θ=11°and the diffraction peak for the corresponding (002) reflection is about 2θ=22°, indicating that the exfoliation process increased the interlayer spacing. d)Tapping-mode atomic force microscopy (AFM) studies revealed that the thickness of GO was of ~1nm and the lateral size of GO was fairly polydisperse, which is characteristic of a fully exfoliated GO sheet.

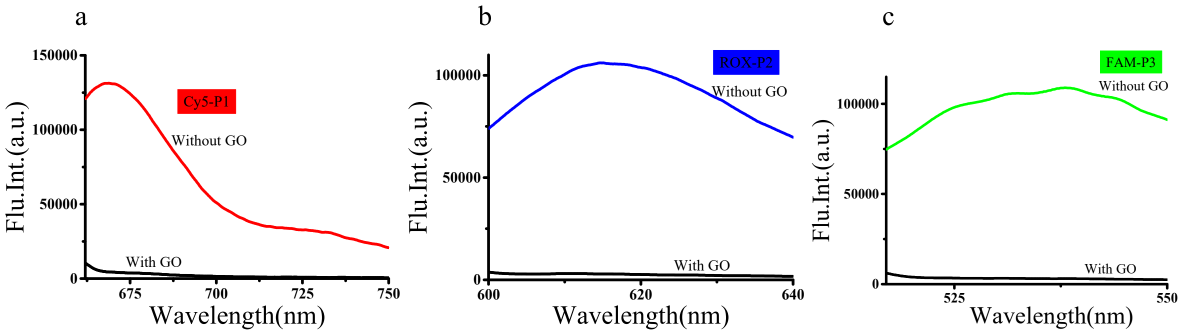

Figure S4. Fluorescence spectra of Cy5-, ROX-, FAM-PNAs with or without GO in HEPES buffer (25 mM, 100 mM NaCl, PH 7.4). Fluorescence of PNA probes were quenched after incubation with GO within 1min.

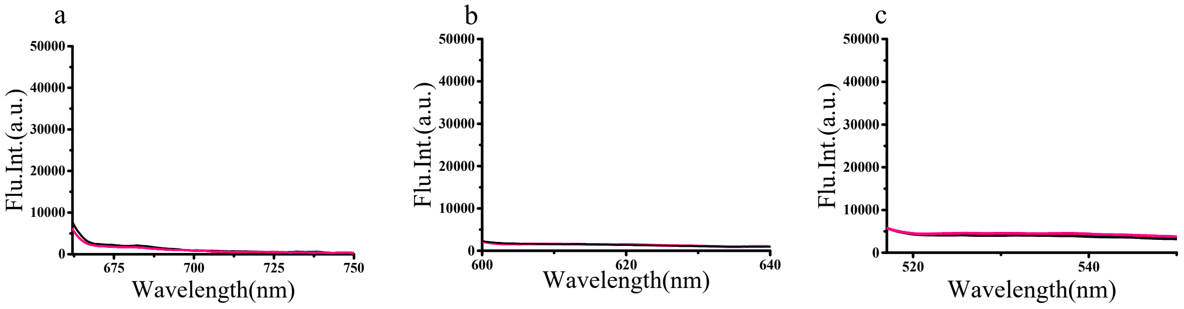

Figure S5. Fluorescence spectra for PNA probes in the presence of GO (black) and incubation with chemically synthesized mRNA with scrambled sequence (purple). No fluorescence signals were observed when excited at 643 nm (a), 575 nm (b), 494 nm (c), which indicated that PNA probes were not hybridized with non-complementary target.
